# A comparison of exercise interventions from bed rest studies for the prevention of musculoskeletal loss

**DOI:** 10.1038/s41526-019-0073-4

**Published:** 2019-05-08

**Authors:** Nagarjun N. Konda, Rama S. Karri, Andrew Winnard, Mona Nasser, Simon Evetts, Eilis Boudreau, Nick Caplan, David Gradwell, Rochelle M. Velho

**Affiliations:** 1 University of Birmingham, Birmingham, UK; 2 Aerospace Medicine Systematic Review Group (AMSRG), Northumbria, UK; 3 University of Birmingham, Birmingham, UK; 4 Northumbria University, Newcastle upon Tyne, UK; 5 University of Plymouth, Plymouth, UK; 6 SeaSpace Research, Liverpool, UK; 7 OHSU, Portland, USA; 8 Northumbria University, Newcastle upon Tyne, UK; 9 Kings College London, London, UK; 10 University of Warwick, Warwick, UK

**Keywords:** Anatomy, Outcomes research

## Abstract

Musculoskeletal loss in actual or simulated microgravity occurs at a high rate. Bed rest studies are a reliable ground-based spaceflight analogue that allow for direct comparison of intervention and control participants. The aim of this review was to investigate the impact of exercise compared to no intervention on bone mineral density (BMD) and muscle cross-sectional area (muscle CSA) in bed rest studies relative to other terrestrial models. Eligible bed rest studies with healthy participants had an intervention arm with an exercise countermeasure and a control arm. A search strategy was implemented for MEDLINE. After screening, eight studies were identified for inclusion. Interventions included resistive exercise (RE), resistive vibration exercise (RVE), flywheel resistive exercise, treadmill exercise with lower body negative pressure (LBNP) and a zero-gravity locomotion simulator (ZLS). Lower limb skeletal sites had the most significant BMD losses, particularly at the hip which reduced in density by 4.59% (*p* < 0.05) and the tibial epiphysis by 6% (*p* < 0.05). Exercise attenuated bone loss at the hip and distal tibia compared to controls (*p* < 0.05). Muscle CSA changes indicated that the calf and quadriceps were most affected by bed rest. Exercise interventions significantly attenuated loss of muscle mass. ZLS, LBNP treadmill and RE significantly attenuated bone and muscle loss at the hip compared to baseline and controls. Despite exercise intervention, high rates of bone loss were still observed. Future studies should consider adding bisphosphonates and pharmacological/nutrition-based interventions for consideration of longer-duration missions. These findings correlate to terrestrial bed rest settings, for example, stroke or spinal-injury patients.

## Introduction

Space exploration programmes anticipate that human missions to Mars will happen within the first half of this century.^1,2^ However, one major obstacle in the future of these missions is musculoskeletal deconditioning.

Recent exercise interventions used on the International Space Station (ISS) are improving in their ability to mitigate musculoskeletal loss, relative to previous interventions that led to ≥10% loss after long-duration space missions.^3–5^ However, these exercises required large devices that are unlikely to be of significant use in small capsular space vehicles for Mars missions.

Previous space missions have reported that the greatest degree of deconditioning is localised to the lower limbs in microgravity.^6^ In particular, bone resorption in the trabecular compartment is higher than in the cortical compartment after 6 months in space.^3,7^ This pattern of bone loss is reflected in osteoporotic patients.^8^

Current theories for microgravity induced bone loss suggest that normal remodelling rates are disturbed, causing bone resorption (osteoclast activity) to occur at a faster rate than ossification (osteoblast activity).^6^ Wolff’s law states that bones adapt according to the loads placed upon them. Decreased stress on bones, due to no gravitational loading on the lower limbs, causes more resorption and less preservation in microgravity.^9^ Fracture risk from reduced bone mineral density (BMD) could, therefore, be a significant problem on Mars missions unless bone degradation is attenuated en-route.^10^ Whilst post-mission fracture risk might be low on return to Earth, it could be mission critical on a Mars mission where direct medical care will not be available.

Other factors that contribute to bone loss include reduced muscle load, reduced immune response and radiation.^11–14^

Physical activity and weight bearing exercises attenuate musculoskeletal degeneration in astronauts and terrestrial groups (e.g. post-operative orthopaedic patients).^15,16^ At present, the Advanced Resistive Exercise Device (ARED) is used on the ISS in combination with a treadmill and cycle-ergometry exercises to simulate free weight exercises.^17^ The ARED device provides a resistive load of 600 lbs compared to 300 lbs used in the previous Interim Resistance Exercise Device (iRED).^4^ AREDs show a trend towards attenuated strength loss compared to iREDs, and an increased bone formation as assessed by serum biomarkers.^4,18^ Peripheral quantitative computed tomography (pQCT) data revealed that BMD was still decreased at the proximal femur and femoral neck regions, despite 12 months of reambulation on Earth.^1^

To further enhance the retardation of bone loss, studies have explored the idea of incorporating vibration into the exercise regimes. These resistive vibration exercises (RVE) aimed to improve muscle power and decrease rates of bone resorption, thus reducing bone loss during bed rest.^19,20^

One challenge of human spaceflight research is to simulate the impact of microgravity on Earth, in order to determine the most cost-effective and practical interventions available. This is commonly accomplished through long duration head-down tilt bed rest studies, which have been shown to be a reliable simulation model.^21,22^ An alternative model to bed rest is dry immersion, which involves immersing the participant in water with an elastic waterproof membrane.^23,24^ The main drawback is that dry immersion studies typically last between 3 and 7 days and rarely beyond 56 days, which is a significantly shorter duration than most bed rest studies.^24^

Although bed rest does not eliminate the influence of gravity, many of the physiological changes that occur mimic those that happen naturally in space such as the absence of work done against gravity, reduced energy requirements and reduced sensory stimulation.^25^ While bed rest fails to remove a Gx (chest to back) loading vector, it is currently considered the most valid method for simulating physiological effects associated with low gravity and to investigate potential countermeasures on Earth.^26^ A 6-degree head down tilt is now used because the resulting cephalad fluid shift is representative of the shift seen in space.^26,27^

Bed rest studies offer a terrestrial analogue for spaceflight where environmental variables can be rigorously controlled, which includes the participants diet. Furthermore, there is a long follow-up period to assess for occupational risk of fractures in later life, an important consideration for astronauts where there is a risk of cumulative skeletal deconditioning.

The primary aim of this review was to investigate the efficacy of exercise countermeasures on attenuating musculoskeletal deconditioning during long duration bed rest when compared to a control arm, in healthy participants.

In addition, this review will discuss the extrapolation of these results to terrestrial patient groups, as musculoskeletal deconditioning is also observed in patients with limited mobility. These include post-operative patients, those with neurological injuries or patients in a critical care setting. This will facilitate discussions around the optimum rehabilitation schedule that incorporates various exercise interventions to address the recovery of different muscle and bone groups.

## Results

### Study characteristics

Eight studies were included for analysis based on the inclusion and exclusion criteria (Table 1). Exercise countermeasures within studies varied and consisted of resistive exercise (RE), RVE, flywheel resistive exercise (FW), treadmill exercise with lower body negative pressure (LBNP) and a zero-gravity locomotion simulator (ZLS).

**Table 1:** Relevant studies for inclusion

Study	Country	Outcome Measure (Imaging)	Intervention (*n*)	Control (*n*)	Length of bed rest (days)	Head-tilt angle	Age	Gender	BMI
Cavanagh^28^	USA	BMD (pQCT) & Muscle Volume (MRI)	Zero Gravity Treadmill (5)	No exercise (6)	84	−6	30.2	6 M 5 F	25.1
Belavy^33^	Germany	BMD (HR-pQCT)	RVE (7) & RE (8)	No exercise (9)	60	−6	32	M	25.7
Rittweger^29^	Germany	BMC (DXA & pQCT) & mCSA (pQCT)	RVE (10)	No exercise (10)	56	0	33	M	23.7
Zwart^31^	USA	BMD (DXA)	Treadmill LBNP (7)	No exercise (7)	30	−6	24	F	n/a
Shackelford^5^	USA	BMD (DXA) & Muscle Volume (MRI)	RE (9)	No exercise (16)	119	0	32	16 M 9 F	24.6
Rittweger^30^	France	BMC & mCSA (pQCT)	FW (9)	No exercise (9)	90	−6	32	M	23.3
Smith^32^	France	BMD (DXA)	Treadmill LBNP & RE (8)	No exercise (8)	60	−6	32	F	21.5
Armbrecht^35^	France	BMD (HR-pQCT)	Treadmill LBNP & RE (8)	No exercise (8)	60	−6	32	F	21.5

*RE* resistive exercise, *RVE* resistive vibration exercise, *LBNP* lower body negative pressure, *FW* flywheel, *pQCT* peripheral quantitative computed tomography, *MRI* magnetic resonance imaging, *DXA* dual energy X-ray absorptiometry, *HR-pQCT* high resolution peripheral quantitative computed tomography

For the intervention and control columns, the *n* refers to number of participants in each arm

1. Cavanagh et al.^28^: This study used a ZLS to evaluate the changes in bone structure with 84 days of bed rest in male and female participants. The ZLS suspended participants horizontally and tethered them to the treadmill via a pneumatic load device that was axially loaded relative to their body to simulate gravity (Supplementary Fig. 1). The load was individually adjusted to replace their normal daily load. BMD data were evaluated both at the spine and hip regions using pQCT. Muscle volume data were assessed at the gastrocnemius and quadriceps. Two-sample *t*-tests were used to analyse the differences between baseline and post-bed rest, and between intervention and control groups.

2. Rittweger et al. (Berlin BedRest Study)^29^: This study used RVE in 56 days of bed rest with male participants (Supplementary Fig. 2). The RVE group used the Galileo Space training device twice daily for 5 days per week. Resistive force was exerted by elastic springs simulating gravitational acceleration. Squats, heel raises, toe raises and kicks were the dynamic exercises performed. Vibration frequency initially started at 19 Hz and ended at 30 Hz. BMD data were presented for four anatomical regions of the tibia and two regions of the radius. Bone mineral content (BMC) data were also measured at these regions as well as at the hip and lumbar spine. Muscle cross-sectional area (muscle CSA) was analysed at the calf and forearm by pQCT. Paired *t*-test (significance between baseline and post-bed rest) and ANOVA (significance between groups) with Bonferroni correction were used to analyse the data.

3. Rittweger et al. (The LTBR Study)^30^: This study conducted a 90-day bed rest with male participants to explore the effect of rotating flywheels which provide resistance in both concentric and eccentric actions (Supplementary Fig. 3). Training sessions with the FW device consisted of a supine squat and calf press and were performed once every 3 days. BMC data were assessed at four regions of the tibia and two regions of the radius. Muscle CSA was measured at the calf and forearm. Paired *t*-test (significance between baseline and post-bed rest) and ANOVA (significance between intervention and control groups) with Bonferroni correction were used to analyse the data.

4. Zwart et al.^31^: Aerobic exercise as opposed to RE was employed by Zwart et al. with female identical twins in 30 days of bed rest.^19^ LBNP treadmill exercise was performed 6 days a week for 40 min per session at an intensity of 40–80% pre-bed rest oxygen consumption (Supplementary Fig. 4). BMD was provided for four regions of the lower body. The LBNP chamber had a sealed flexible waist seal. ANOVA (significance between baseline and post-bed rest and between intervention and control groups) with Bonferroni correction was used to analyse the data.

5. Smith et al. (WISE 2005 Study)^32^: This study combined the use of LBNP treadmill with RE in women during 60 days of bed rest. RE consisted of leg press and calf press with an inertial ergometer. Treadmill exercise lasted for 40 min per session at varying intensities, 3 to 4 days per week. Treadmill and RE were performed on separate days. Results presented were BMD at the trochanter, hip, leg, femoral neck and spine. ANOVA (significance between baseline and post-bed rest and between intervention and control groups) with Bonferroni correction was used to analyse the data.

6. Shackelford et al.^5^: This study used a horizontal exercise machine which combined a cabled pulley system with weight plates to achieve RE training. The exercise machine was used by the intervention group for 6 days per week during 17-week bed rest period to exercise the upper and lower body, 3 days each on alternate days. Bed rest was horizontal for this cohort of participants, which included males and females. BMD data was presented as a percentage change from pre to post-bed rest at the lumbar spine, femoral neck, trochanter, total hip, calcaneus, distal radius and proximal radius. A paired *t*-test was used to identify significance between baseline and post-bed rest and analysis of covariance (ANCOVA) was used for effects between intervention and control groups.

7. Belavy et al. (2^nd^ Berlin BedRest Study)^33,34^: This study had three trial arms in which participants either performed RE, RVE or acted as a control during a 60-day bed rest period. Exercise training, including lower body exercises such as bilateral squats, single and double leg heel raises, was performed three times a week during bed rest. The Galileo space exercise device was used, like the primary Berlin Bedrest study.^29^ The frequency of vibration varied between each exercise between 16 and 26 Hz. Both exercise groups performed the same workouts except the RVE group that had whole-body vibration applied simultaneously. HR-pQCT data were reported for the distal tibia and distal radius and ANOVA was used to determine effects between baseline and post-bed rest, and between intervention and control groups.

8. Armbrecht et al. (WISE 2005 Study)^35^: This study used the same protocol and participants as the WISE 2005 study.^32^ However, this study used HR-pQCT to measure changes in trabecular and cortical bone density as opposed to dual X-ray absorptiometry (DXA) as used by Smith et al.^32^ ANOVA was used to determine effects between baseline and post-bed rest and between intervention and control groups.

### BMD and BMC comparisons

#### Effects of bed rest at different anatomical regions

Six out of eight studies provided BMD data measured by DXA or pQCT (Table 2), with the spine and hip being the most commonly reported scan locations. In four out of five studies, the most significant losses were observed at the hip region, with significantly greater losses in the control arm compared to the intervention arms.^5,28,31,32^ Cavanagh et al.^28^ and Zwart et al.^31^ reported a significant increase in hip BMD post-bed rest with an exercise intervention. Although both losses and gains were reported in the spinal and femoral neck regions, the changes were not statistically significant. However, Shackelford et al.^5^ reported a statistically significant loss at the spine for the control group but a significant gain for the intervention group.

**Table 2:** Comparative results of the % change in Bone Mineral Density/Bone Mineral Content at 7 skeletal sites

Study	Post bed-rest data measured at	Group	Spine	Total Hip	Distal tibia diaphysis	Tibia epiphysis	Proximal radius	Distal radius	Femoral neck
Cavanagh^28^	PBR	CON (*n* = 6)	1.82	−4.59*	x	x	x	x	−4.09
		ZLS (*n* = 5)	0.84	1.34*^	x	x	x	x	−0.14
Rittweger^29^	PBR + 14	CON (*n* = 10)	−1.29	−1.39	−0.39*	−3.60*	−0.06	−0.32	x
		RVE (*n* = 10)	0.12	0.00	0.02	−0.36	0.00	−0.08	x
Zwart^31^	PBR	CON (*n* = 7)	x	−1.64**	−0.44	x	x	x	−0.10
		LBNP TM (*n* = 7)	x	0.31**^	−0.53	x	x	x	1.99
Rittweger^30^	PBR + 14	CON (*n* = 9)	x	x	−1.60*	−6.00*	−0.60	−0.40*	x
		FW (*n* = 9)	x	x	−0.9*^	−2.80*	−0.70	−0.40*	x
Smith^32^	PBR	CON (*n* = 8)	0.30	−4.03*	−1.25*	x	x	x	−1.13
		LBMP TM + RE (*n* = 8)	1.14	−1.99*^	−0.33*^	x	x	x	0.12
Shackelford^5^	PBR	CON (*n* = 18)	−1.30*	−3.40*	x	x	−0.20	0.00	−1.50
		RE (*n* = 9)	3.40*^	−0.90*^	x	x	0.20	−1.00	0.10

Comparative results of the % change in bone mineral density/bone mineral content from pre-bed rest values at 7 skeletal sites

*COn* control group, *ZLS* zero-gravity locomotion simulator, *RVE* resistive vibration exercise, *LBNP TM* lower body negative pressure treadmill, *FW* flywheel, *RE* resistive exercise

* Indicates *p* < 0.05 compared to baseline, ** indicates *p* < 0.01 significance from baseline (Cavanagh^28^ used two-sample *t*-test. Zwart^31^ and Smith^32^ used post hoc testing with Bonferroni method. Rittweger^19^ and Shackelford^5^ used paired *t*-test), ^ indicates *p* < 0.05 significance between exercise and control groups (Cavanagh^28^ used two sample *t*-test. Shackelford^5^ used analysis of covariance. All other studies used analysis of variance). For the group column, *n* is the number of people in each study arm. x represents unavailable data. PBR + number indicates number of days post-bed-rest BMD data was measured at

The distal tibia was shown to have statistically significant losses in three out of four studies.^29,30,32^ Two of these studies also indicated statistically significant differences between control and intervention groups.^30,32^

**Fig. 1:**
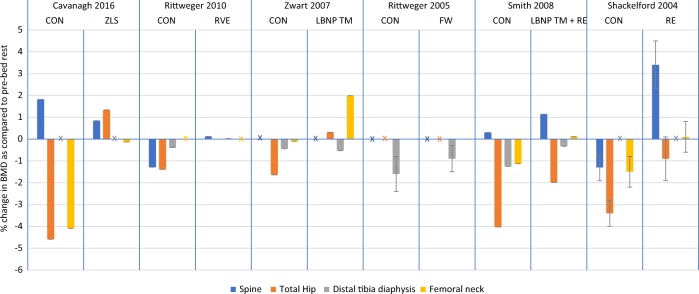
Results from the 6 included studies for the spine, hip and distal tibia diaphysis and femoral neck. Changes in BMD are expressed as mean % change from pre-bed rest (SD). Data from intervention and control arms are stated. X refers to absent data

There were also significant losses (*p* < 0.05) in the control arm at the tibial epiphysis (consisting of trabecular bone) of 3.6 and 6% from the studies by Rittweger et al.^29^^[,30^ respectively. Following this, significant differences were observed between study arms at the tibia epiphysis by Rittweger et al.^30^ (*p* < 0.05) (Fig. 1). Only Rittweger et al.^30^ reported a decrease in the distal radius BMD from baseline (*p* < 0.05). There were no significant differences between the study arms at the proximal and distal radius sites.

#### Effects of exercise intervention

RE had the largest effect on BMD in the spinal region. Shackelford et al.^5^ reported an increase of 3.4%. No other intervention showed a statistically significant improvement from the control group in the spine. Shackelford et al.^5^ also showed RE to be significantly different to the control group with respect to the hip, where the loss in BMD decreased from 3.4 to 0.9%.

Participants that were exposed to RE combined with LBNP treadmill had a significant decrease of 1.99% in BMD relative to a 4.03% loss in the control arm (*p* < 0.05).^32^ The largest impact resulting from using the ZLS in the Cavanagh et al. study where the participants gained BMD at the hip by 1.34%. LBNP treadmill also showed an increase by 0.31% at the hip compared to −1.64% in the control group.^28^

**Fig. 2:**
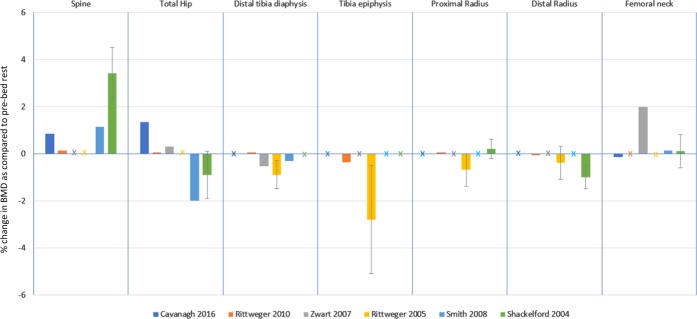
Intervention results from the 6 included studies to compare which interventions have greater effectiveness for a specific skeletal site. Changes in BMD are expressed as mean % change from pre-bed rest (SD). X refers to absent data

Figure 2 compares different exercise intervention groups. FW and LBMP treadmill + RE both showed significant differences from the control group at the distal tibial epiphysis.^29,30^ There were no significant differences between exercise and control groups at radial and femoral neck sites.^29,32^

### HR-pQCT analysis

Belavy et al.^33^ and Armbrecht et al.^35^ measured BMD at the distal tibia and distal radius exclusively using HR-pQCT (Table 3). The participants in the study by Belavy et al.^33^ were male and the participants in Armbrecht et al.^35^ were women.

**Table 3:** Comparative results from studies that used HR-pQCT, measured 3 days post-bed-rest

Study	Group	Distal tibia			Distal radius		
		Total density	Cortical density	Trabecular density	Total density	Cortical density	Trabecular density
Belavy^33^	CON (*n* = 9)	−1.20~	−0.40*	−0.60*	0.2	0	0
	RVE (*n* = 7)	−1.20**	−0.4	−0.9	0.5	0.2	0.60*
	RE (*n* = 8)	−1.10~	−0.40*	−0.70**^	0.2	0.2	−0.6
Armbrecht^35^	CON (*n* = 8)	−2.50**	−0.90**	−3.80*	−0.4	−0.2	−0.50*
	RE + LBNP TM (*n* = 8)	−1.50~	−0.40**	−2.40~	−0.4	−0.2	−0.80*

Comparative results from studies that used high resolution peripheral quantitative computed tomography (HR-pQCT), measured 3 days post-bed-rest. The % change in total, cortical and trabecular density from pre-bed rest values is displayed at the distal tibia and radius

*CON* control group, *RVE* resistive vibration exercise, *RE* resistive exercise, *LBNP TM* lower body negative pressure treadmill

* Indicates *p* < 0.05 compared to baseline, ** indicates *p* < 0.01 compared to baseline, ~indicates *p* < 0.001 significance between baseline (analysis of variance testing), ^ indicates *p* < 0.05 significance between exercise and control groups (analysis of variance testing). For the group column, *n* is the number of people in each study arm

#### HR-pQCT changes at the distal tibia

Both studies showed significant losses at the distal tibia in all study arms (*p* < 0.01).^33,35^ Groups in both studies exhibited a greater trabecular loss compared to cortical bone loss.

Belavy et al.^33^ showed significant decreases from baseline (*p* < 0.01) in BMD for all three trial arms (1.1% RE, 1.2% RVE and 1.2% control). They also showed significant changes from baseline for control and RE groups for cortical density (−0.4% for both groups), and trabecular density (−0.6% control and −0.7% RE). The only significant difference between control and intervention group was reported in the trabecular density where the loss in BMD was significantly greater in the intervention group (0.7%) than in the control group (0.6%).

Armbrecht et al.^35^ reported greater losses than Belavy et al.^33^ in both groups at the distal tibia. Total density significantly reduced by 2.5% (*p* < 0.01) in the control group and by 1.5% in the RE plus LBNP treadmill group (*p* < 0.001). The trabecular density reduced by 3.8% for the control group (*p* < 0.05) and 2.4% in the RE plus LBNP treadmill group (*p* < 0.001). Cortical bone loss was less substantial with losses of 0.9 and 0.4% for control and intervention arms respectively (*p* < 0.01).

#### HR-pQCT changes at the distal radius

The distal radial trabecular density was the greatest affected area. Belavy et al.^33^ reported that RVE exercise improved trabecular density by 0.6% relative to baseline (*p* < 0.05). In contrast, Armbrecht et al.^35^ showed a statistically significant decrease in trabecular density from baseline.

There were no significant differences between study arms. There were also no significant changes in total and cortical density at the distal radius in either study.

### Muscle comparisons

Rittweger et al.^29,30^ reported muscle CSA at the calf and forearm, whilst Shackelford et al.^5^ and Cavanagh et al.^28^ reported muscle volume changes for the calf and quadriceps (Table 4).

**Table 4:** Muscle cross-sectional area results expressed as % change from pre-bed rest

Study	Group	Anatomical Location (% muscle CSA change from pre-BR)		
		Calf	Forearm	Quadriceps
Rittweger^29^	CON (*n* = 10)	−18.0**	−1.0	x
	RVE (*n* = 10)	−8.0**^	−0.6	x
Rittweger^30^	CON (*n* = 9)	−25.6*	−6.4*	x
	FW (*n* = 9)	−17.3**^	−7.6*	x
Cavanagh^28^	CON (*n* = 6)	−33.0**	x	−23.3**
	ZLS (*n* = 5)	−13.8**^	x	−7.2**^
Shackelford^5^	CON (*n* = 16)	−28.1*	x	−15.8*
	RE (*n* = 9)	−6.6*^	x	2.0^

Muscle cross-sectional area (muscle CSA) results expressed as % change from pre-bed rest. Rittweger et al.^29,30^ measured muscle CSA one day before the end of the bed-rest period. Cavanagh et al.^28^ and Shackelford et al.^5^ measured muscle volume or CSA at the completion of bed-rest

*CON* control group, *RVE* resistive vibration exercise, *FW* flywheel, *ZLS* zero-gravity locomotion simulator, *RE* resistive exercise

* Indicates *p* < 0.05 compared to baseline, ** indicates *p* < 0.01 significance from baseline (Cavanagh^28^ used a two sample *t*-test. All other authors used a paired *t*-test), ^ indicates *p* < 0.05 significance between exercise and control groups (Cavanagh^28^ used a two sample *t*-test. Rittweger^29,30^ used analysis of variance. Shackelford^5^ used analysis of covariance)

**Fig. 3:**
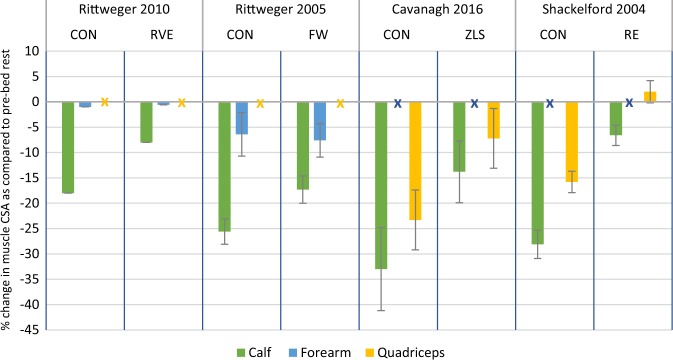
Results from the four included studies for the muscle changes at the calf, forearm and quadriceps. Changes in muscle CSA are expressed as mean % change from pre-bed rest (SD). Data from intervention and control arms are stated. X refers to absent data

There was a 33% decrease in the control group calf measurements (*p* < 0.01) (Fig. 3).^30^ All exercise interventions significantly attenuated the loss of calf compared to controls (*p* < 0.05). A greater loss of muscle in the calf was associated with a greater reduction in BMD in the distal tibial diaphysis in the control and exercise groups of both Rittweger studies.^29,30^ Consequently, there was less bone and muscle lost by the intervention groups than the controls.^29,30^

Muscle volume and CSA also significantly decreased at the quadriceps as reported by Cavanagh et al.^28^ and Shackelford et al.^5^ respectively. These losses were not as great as that of the calf muscle, with −23.3% being the greatest decrease in muscle area in the control group (*p* < 0.01).^28^ The exercise interventions resulted in significantly less muscle mass lost.

Only Rittweger et al.^30^ reported significant losses at the forearm, with −6.4% for the control and −7.6% for the intervention group. There was no significant difference between trial arms.

### Bone biomarkers

Table 5 shows the changes in biomarkers as observed by the studies. Three studies did not provide any bone marker data.^29,33,35^ The greatest changes were observed in the bone formation markers excreted in the urine. Three out of four studies showed significant increases from baseline in n-telopeptide (NTX) and deoxypyridinoline (DPD) and all the studies showed significant increases in pyridinium cross-links (PYD) from baseline. However, the changes were not significant between the intervention and control groups.

**Table 5:** The biomarker changes from baseline to post-bed-rest

Study	Duration (Days)	Group	Bone formation markers			Bone resorption markers			Other key markers					
			NTX	DPD	PYD	BSAP	Total ALP	OCN	Urine Calcium	Serum Calcium	PTH	1,25(OH)_2_ Vit D	25(OH) Vit D	Creatinine
Shackelford^5^	119 days	CON	↑*	↑*	↑*	↓	↑	↑	x	↑	↓	↓*	↑	x
		RVE	↑*	↑*	↑*	↑*^	↑*^	↑*^	x	↓*	↓	↑^	↑	x
Rittweger^30^	90 days	CON	x	↑*	↑*	x	↑**	x	x	=	=	x	x	x
		FW	x	↑*	↑*	x	↑**	x	x	=	=	x	x	x
Smith^32^	60 days	CON	↑	=	x	↑	↑	=	=	↑	↓	↓	↓*	x
		LBNP TM + RE	↑	↑	x	↑	↑*^	↑	↓	↓	↓	↓	↓*	x
Zwart^31^	30 days	CON	↑**	↑**	↑**	↓	↓	↓	↑	↓**	↓	↓	↓	↑*
		LBNP TM	↑**	↑**	↑**	↓	↓	↑	↓	↓**	↓	↓	=	↑*
Cavanagh^28^	84 Days	CON	↑*	x	x	↑	x	x	↑	x	x	x	x	↓
		ZLS	↑*	x	x	↑	x	x	↓	x	x	x	x	↓

The biomarker changes from baseline to post-bed-rest. ↓ decrease in levels, ↑ increase in levels, X no data, = no change

*NTX* n-telopeptide, *DPD* deoxypyridinoline, *PYD* pyridinium cross-links, *total ALP* total alkaline phosphatase, *BASP* bone specific alkaline phosphatase, *OCN* osteocalcinin, *PTH* Parathyroid hormone, *CON* control group, *RVE* resistive vibration exercise, *FW* flywheel, *LBNP TM* lower body negative pressure treadmill, *ZLS* zero-gravity locomotion simulator

*Significant from baseline *p* < 0.05, **significant from baseline *p* < 0.01 (Cavanagh^28^ used a two sample *t*-test. Zwart^31^ and Smith^32^ used post hoc testing with the Bonferroni method. Rittweger^30^ and Shackelford^5^ used analysis of variance), ^significant between exercise and control groups *p* < 0.05 (Cavanagh^28^ used two sample *t*-test. All other studies used analysis of variance)

With respect to the bone resorption biomarkers, total alkaline phosphatase (ALP) was the most prominent biomarker and was significantly increased in three of the four studies.^5,30,32^ Additionally, both Shackelford et al.^5^ and Smith et al.^32^ showed significant changes between control and intervention groups. Shackelford et al. also showed statistically significant changes from baseline and between groups for bone specific alkaline phosphatase (BSAP) and osteocalcinin (OCN). The changes in BSAP and OCN were not significant in the other studies.^5^

Zwart et al.^31^ and Shackelford et al.^5^ showed significant decreases in serum calcium in their intervention groups. Only Shackelford et al.^5^ reported significant changes in 1,25(OH)_2_ Vitamin D, with the control group showing a significant decrease in levels that was also significantly different to the intervention group in which levels increased. Smith et al.^32^ showed significant decreases in 25(OH) Vitamin D levels from baseline. Creatinine levels were reported by Zwart et al.^31^ to be significantly higher after bed rest.

## Discussion

This review evaluated eight papers that investigated bone loss at several skeletal sites. The studies indicated significant losses in both control and intervention groups post bed rest. The lower limb skeletal sites demonstrated the greatest losses. The entire hip showed greatest changes in BMD with up to −4.59% (*p* < 0.05) and −6% (*p* < 0.05) at the tibial epiphysis.^28,30^ Exercise interventions had the greatest effect in hip region, with Cavanagh et al. and Zwart et al. both reporting gains in BMD at the hip of 1.34 and 0.4% respectively (*p* < 0.05).

Significantly lower rates of bone loss in the exercise groups were observed compared to control groups for lower body skeletal sites. The effect of exercise interventions were less pronounced in the arms compared to lower body sites. This is understandable given that upper limb bones are non-weight bearing. The studies included in this review showed that no significant changes were observed at the proximal radius and only one study (Rittweger et al.^30^ observed significant changes, at the distal radius. This was noted in space-flight by Vico et al.^7^ who studied 11 participants after 6-month MIR flights, and found that the greatest changes were in the load bearing skeleton compared to bone loss at the distal radius. This is also consistent with data from other space-flight missions that show the greatest bone and muscle losses in the lower limb.^36–38^

HR-pQCT data indicated that the RE groups had significant improvements in loss of bone density at the distal tibia.^33,35^ When the LBNP treadmill was combined with RE by Smith et al.,^32^ significant changes between control and intervention groups were observed at the distal tibia. In contrast, Zwart et al.^31^ reported that LBNP treadmill alone caused no significant effects. These results are reflected in the use of the ARED device on board the ISS which requires high-intensity RE. The increased RE in the ARED device compared to the iRED led to a decreased monthly loss in BMD from around 1 to 0.3–0.5% per month.^1^ The LBNP treadmill regimen was similar in terms of intensity for each session in the studies by Zwart et al.^31^ and Smith et al.^32^ However, Zwart et al. performed LBNP treadmill for 6 days per week while participants in Smith et al. only exercised with the treadmill for two to 4 days per week, with RE performed on the other days.^31,32^ This further signifies the positive impact of the addition of RE to treadmill exercise in attenuating bone loss. Other studies suggested significant correlation between treadmill training intensity and loss of muscle mass with up to 59% less muscle lost in high-intensity compared to low-intensity treadmill exercise.^39^ This is evident in this review as Cavanagh et al.^28^ utilised a higher intensity of treadmill exercise and showed less bone mass lost at the hip region in exercise group vs control, compared to the exercise participants in Zwart et al.^31^ vs their control.

It was noted that trabecular bone density decreased more rapidly than cortical bone in both studies using HR-pQCT as the imaging modality. Cortical bone is denser and calcified, while trabecular (spongy) bone is generally thought to have a much higher turnover rate.^40^ Cervinka et al. found that the greatest cortical losses occurred in the first 2 months of bed rest but trabecular losses were greater over a longer time period.^3^ This is reflected in space-flight mission data and long-term immobilisation.^7^

Changes at the spine were non-significant in three out of four studies. Only Shackelford et al.^5^ found a modest decrease in BMD in their control group and a 3.4% increase in their intervention group (*p* < 0.05).^5^ The non-significant changes observed in the other three studies are possibly explained by participants continuously moving throughout the day and consequently exerting moments about their spinal joints during bed rest.^28^ Seven out of eight cosmonauts had between 2.5 and 10.6% decrease in BMD at the lumbar vertebrae post-ISS mission.^41^ This is important as astronauts have an almost 3× increased risk of lumbar and cervical inter-vertebral disc herniation compared to the general population.^42^

The muscle CSA data highlights the impact that bed rest has on the lower limb skeletal sites and the minimal impact this condition has on the upper limb, with only Rittweger et al.^30^ reporting significant losses of 6.4 and 7.6% at the forearm compared to 25.6 and 17.3% at the calf for the control and intervention group, respectively. The greater muscle losses at the calf correspond to the greater bone mass loss at the tibia. Moreover, RE, which had the greatest impact on attenuating bone loss, also had the greatest influence on the attenuation of muscle loss, as demonstrated by a significant increase in muscle CSA from baseline at the quadriceps compared to the control group (2%, *p* < 0.05). Nevertheless, all interventions had a significantly lower loss of muscle CSA compared to control groups at the calf. Although Wolff’s mechanical loading theory suggests that remodelling rates change according to stress placed upon bones, it is important to consider that muscles are essentially coupled to bones.^9^ An individual that produces higher loads on muscle exerts a higher load on bone. This synergistic relationship means that an increase in muscle loading leads to preservation of bone. Hindlimb unloading studies and the injection of Botulinum toxin in animal models have further correlated this close interplay between muscle loss and consequent bone loss.^43^

Individual studies combined difference exercise principles, making it difficult to evaluate which method had the greatest impact on attenuating bone loss. Nevertheless, all the RE interventions utilised exercises that target multiple muscle groups such as full body squats and bilateral heel and toe raises. These had a positive impact on preserving muscle and bone mass. The time spent exercising also varied between studies. Participants in the study by Rittweger et al.^30^ only spent 2 days a week exercising during the bed rest period in contrast to other studies such as Zwart et al. where participants exercised 6 days a week.^29,31^ The supine squat and calf press exercises in Rittweger et al.^30^ had significant effects in decreasing bone loss at the tibia diaphysis and epiphysis. The addition of knee-ups and hip abduction exercises in the study by Shackelford et al.^5^ contributed to decreased bone loss in the hip area.

Studying the bone biomarkers indicated significant increases in bone formation biomarkers in the urine, NTX, DPD and PYD from baseline. No significant changes were observed in bone resorption markers. This was reflected in the data obtained from the ISS which demonstrated that exercise attenuates BMD loss through increased bone formation rather than mitigation of the increased bone resorption.^4^ However, the extent of the impact of exercise countermeasures on bone markers remains unclear. The levels of bone biomarkers are known to vary between different individuals especially with age, but if considered carefully do provide valuable clues for the manner and magnitude of long-term recovery from bed rest and post-space flight.^44^

### Other countermeasures to prevent bone loss

In 2013, the NASA bone summit reported that exercise alone was insufficient to prevent bone loss after space flight.^1^ Other countermeasures have, therefore been more recently investigated. Leblanc et al.^45^ investigated astronauts who spent a mean duration of 5.5 months on board the ISS and compared ARED exercise alone (*n* = 11); ARED plus 70 mg Alendronate weekly (*n* = 7); and iRED exercise alone (*n* = 18). The combination of a bisphosphonate and ARED attenuated losses in BMD at the spine, hip and pelvis as measured by DXA as well as pQCT compartmental losses in trabecular and cortical mass at the hip. Smith et al. reported that the ARED plus Alendronate intervention increased the bone formation markers without affecting bone resorption markers, highlighting the impact of pharmacological adjuncts at mitigating bone loss.^4,32^

Another area of focus is delivering optimum nutrition that can further attenuate bone loss. Heer et al.^46^ investigated the use of a high-protein intake on bone turnover in women after a 60-day bed rest study. The high-protein intake worsened the bed rest induced increase in the bone resorption marker C-telopeptide. Zwart et al.^47^ reported that a higher ratio of dietary animal protein to potassium corresponded to a higher excretion of calcium and markers of bone resorption.

### Limitations

The reporting of BMD and BMC data together was justified as values provided in the results section only reflect the percentage changes in bone loss as compared to baseline data. Therefore, using a conversion score to convert BMD into BMC or vice versa would have no effect on the percent change figure.

Whilst bone markers are greatly impacted by inter-individual variability, imaging modalities provide a more comparable means of bone loss analysis. HR-pQCT is a relatively new method of imaging with a greater ability to compartmentalise the trabecular and cortical segments than pQCT. However, even slight movements during image acquisition can affect the segmentation of bone and the surrounding soft tissue, leading to motion blurring and artefacts.^33^ Automatic and manual motion measurement can improve the quality of image acquisition for large cohort and multi-centre studies.^48^ Future bed rest studies may consider the use of QCT-based finite element modelling (FEM); a technique used to perform structural analysis on complex loading conditions or objects with an irregular geometry.^49^ Previous studies have found the correlations between FEM-predicted strength and measured proximal femoral strength to be stronger than correlations measured using DXA.^50–54^

Astronauts are known to lose bone mass at different rates.^55^ The large variability between participants is difficult to overcome due to the massive ethical and funding issues needed to commit to bed rest studies. This review partially alleviates this limitation by comparing multiple bed-rest studies. However, another limitation surrounds the varied length of the bed rest studies. The shortest analysed was 30 days whilst the longest was 119 days. The amount of bone loss might vary with certain periods of time and so exercise interventions might be more useful at specific time points, which are difficult to determine.

Furthermore, several studies have used female participants and it is well known that gender-specific hormones play a large role in bone metabolism.^56,57^ Armbrecht et al.,^35^ who studied female participants, measured higher losses in BMD than Belavy et al.^33^ at both the distal tibia and the distal radius. Conversely, Morgan et al.^58^ found that men and women do not have substantially different responses to skeletal unloading from the analysis of bone resorption markers post-bed rest.

### Missions to Mars

Whilst it is very difficult to extrapolate the results of bed-rest studies to the duration of a Mars mission, the results of this review provide a background into the bone loss that occurs within shorter bed-rest periods. Furthermore, these results help demonstrate the effectiveness of various exercise interventions available and their impact at different bone sites.

Space-flight data from Smith et al.^59^ suggested that astronauts using ARED on-board the ISS can return from space flight missions with no significant BMD changes compared to baseline. However, the length of the missions this finding was based on was on average 134 +/− 64 days for the ARED group. This poses several issues when considering significantly longer-duration missions. A recent study suggests that a long duration mission to Mars is unlikely to have enough space for a large device such as the ARED.^29,60^ This can be addressed either through the use of alternative countermeasures which use a smaller payload or improving rehabilitation immediately post-mission over a longer period. Recommendations from the NASA bone summit include increased research into pharmacological interventions in ground-based spaceflight analogues, and the use of QCT and FEM rather than DXA for risk surveillance post-spaceflight.^1^

Belavy et al.^33^ provided two-year follow-up data from their HR-pQCT bed-rest studies. This showed a decrease in trabecular density which persisted up to the two-year follow-up point in the recovery phase.^33^ Similarly, Armbrecht et al.^35^ reported incomplete bone density recovery at the distal tibia and distal radius 1 year after bed rest. This can be explained by evidence suggesting that full recovery in cortical bone can take up to 2 years’ post-fracture in some skeletal regions.^5^ Rittweger et al.,^29^ using conventional pQCT, revealed a full recovery at metaphyseal and diaphyseal sites, but not at the epiphysis. This highlights the potential risks of a longer Mars mission which can last up-to 520 days where recovery time to normal bone density could be far longer.^61^

It is important to recognise other consequences of bone loss, including the increased risk of kidney stones. Smith et al.^62^ reported an increased risk of sodium urate stones compared to the general population in a study of 42 astronauts. Furthermore, loss of strength could limit the ability of the crew to perform physical operational tasks upon arrival on Mars in a hypogravitational environment.

### Future recommendations

The mean number of participants in the eight bed rest studies included in this review was 18 (range: 11–25) and the mean bed rest period was 70 days (range: 30–119). Due to the large interindividual variability and sex-related changes, future bed rest studies should incorporate larger sample sizes and consider extended periods of bed rest to simulate long duration missions.

Both Rittweger et al.^30^ and Armbrecht et al.^35^ found significant, albeit small, decreases in the distal radius bone mass after bed rest in the exercise groups. This may be explained due to neither of the studies performing targeted upper limb exercises. Although it is evident that the lower limb changes are more pronounced, future exercise regimes should still mitigate upper limb muscle and bone losses with targeted exercises.

Although combination with bisphosphonates such as alendronate or zolendronic acid show promising results, there are limited studies that combine pharmacological interventions with exercise in bed rest, let alone investigating pharmaceutical countermeasures at different dosages.^4^ Other anti-resorptives such as Teriparatide (exogenous parathyroid hormone), which is indicated for post-menopausal women with osteoporosis at high risk of fracture, should be investigated.^1,60^ Teriparatide may be used intermittently in the event of an in-flight fracture combined with low-intensity pulsed ultrasound (LIPUS) which can have a positive effect on fracture healing.^63^ Denosumab is a human monoclonal antibody that inhibits osteoclast formation, function, survival and, ultimately, decreases bone resorption. This drug requires longer-term clinical testing but may be an option in the future to attenuate bone loss.^1^

The use of medication is accompanied by the risk of adverse events. Therefore, an important consideration is to optimise the nutrition schedule. Future studies should investigate the combination of exercise with different nutritional regimes.

Many astronauts experience back pain and disc herniation due to weakening of specific lumbopelvic muscles.^42^ There is scope for new inventions such as the Functional Re-adaptive Exercise Device (FRED) that uses an adapted elliptical trainer to target these muscles and aid recovery.^64^ It is important to consider rehabilitation devices that target specific muscles which also have secondary effects on bone.

### Impact on ground-based medicine

The analysis of exercise countermeasures is not limited to treating astronauts during space flight. Long periods of inactivity and bed rest are experienced by patients who have had, for example, a stroke, coma or spinal cord injury. These patients are different from the patients in bed rest studies, and require rehabilitation that is not highly intensive and is able to target specific muscle and bone groups.^65^ Dauty et al.^66^ found that the loss of bone mass is greatest in the lower limb (70% in the distal femur and 52% in the proximal tibia) in spinal cord injury patients. Such patients with paralysis would have to rely on vibration technology which simulates neuromuscular activation and induces mechanical stress on bone.^67^ Research is still lacking in the quantity and timing of physical activity for stroke patients.^15^ The next step is to tailor these interventions for patients to provide a fast recovery and prevent disuse osteoporosis.

Bed rest significantly decreased BMD at the hip and tibia particularly affecting trabecular bone. The bed rest period did not have a significant effect on bone loss at the upper body sites. The ZLS, LBNP and RE significantly attenuated bone loss at the hip from baseline and between intervention and control groups. Significant muscle losses were reported at the calf and quadriceps post-bed rest, which were attenuated by RE, FW and ZLS. The most common exercise utilised within RE interventions included those that target multiple muscle groups, for example, the full body squat and bilateral heel and toe raises. Future bed rest studies should consider the implementation of exercises to target isolated muscles such as hip abductors, hamstrings and lumbopelvic muscles in addition to the core exercises.

Further ground-based research is needed to investigate the combination of exercise and pharmaceutical interventions along with longer bed rest periods and larger cohort sizes. The addition of nutritional and pharmacological countermeasures may aid in attenuating bone loss, but further refinement is needed to existing exercise countermeasures. The mass and volume constraints of current exercise countermeasures warrants modification or redesign for long duration missions.

## Methods

### Eligibility criteria

Eligible bed rest studies include participants aged 19–50. Suitable studies had healthy participants with no comorbidities such as diabetes, osteopathic or cardiovascular diseases that could impact bone turnover rates or affect exercise capacity.

Studies for inclusion had to be randomised controlled trials (RCTs) assessing the effectiveness of at least one intervention. All included studies had to include an intervention arm with only exercise interventions and no combination with pharmacological treatments. The intervention and control groups had to be treated equally in all other aspects of the trial to prevent performance bias.

The primary outcome was to evaluate musculoskeletal changes after bed-rest with different exercise interventions. Therefore, studies were excluded from review if they didn’t provide BMD/BMC or muscle CSA/volume data from DXA imaging or pQCT. These two methods of imaging are commonly used to measure bone density. Both imaging techniques are able to accurately calculate BMD in the trabecular and cortical compartments.^68^ Limiting results to two common specific imaging methods made results more comparable between studies.

Although bed rest fails to take out a Gx force vector, it is considered the most valid Earth based simulation for reproducing the deconditioning caused by microgravity exposure in many human physiological systems.^69^

The review was limited to English language studies due insufficient resources to interpret other languages. Unpublished studies were considered for inclusion.

### Study selection

Electronic searches were conducted using MEDLINE (In-Process & Other Non-Indexed Citations) and The Cochrane Library utilising search strategies as in supplementary table 1.

Two independent authors (NK and RK) selected relevant studies by filtering the abstracts (*n* = 133). The studies were then compared against the eligibility criteria looking for bed rest RCTs with an exercise intervention (*n* = 13). The studies by Yang et al.^44^, Smith et al.^70^ and Belavy et al.^71^ were excluded because BMD or BMC data were not available. The paper by Berg et al.^72^ did not publish any numerical muscle CSA data and therefore made it difficult to compare the results. The study by Wang et al.^73^ was excluded due to inconsistencies between the text, figure and tables in the results section. The full texts that meet the inclusion criteria are presented in Table 1 (*n* = 8).

The paper by Belavy et al.^33^ used high resolution peripheral computed tomography (HR-pQCT) and was the first study to use this imaging technique to measure exercise with inactivity. Armbrecht et al.^35^ also used HR-pQCT. As there is weaker correlation between HR-pQCT and pQCT compared to the strong correlation between DXA and pQCT, these two studies have been analysed separately.^74^

Biochemical markers were measured by five of the eight studies. Our review presents these results as increases or decreases from baseline with no figures due to the following factors. All the studies presented the values with differing units of measure. Rittweger et al.^30^ only presented the results graphically and did not provide the values.^29^ Cavangh et al.^28^ presented the results as changes in values from baseline. Shackleford et al.^5^ gave % changes from baseline.

### Risk of bias

The risk of bias was evaluated using the Cochrane Risk of Bias Tool by two independent authors (NK and SK) (Supplementary table 2).^75^ Included studies were scored as either high, low or unclear risk of bias. High risk of bias in outcome reporting was present in two of the studies where not all imaging data were provided. For example, the study by Zwart et al.,^31^ the DXA scanning was performed at 12 skeletal sites but results for only five regions was provided. Rittweger et al.^30^ performed scans at five time periods during the study but results of only two time periods were provided in the results section.^29^ It was a similar scenario with the papers by Smith et al.,^32^ Armbrecht et al.^35^ and Cavanagh et al.,^28^ where results from all specified time points were not provided. It was unclear in all studies whether all primary and secondary outcomes were reported in a pre-specified way. Four out of eight studies failed to specify whether compliance was monitored throughout bed-rest with camera or video recording. All but one of the studies randomised participants. Shackelford et al. described allocating participants to the intervention or control arm based on order of application, therefore, being high risk for selection bias. Blinding of participants and personnel was difficult to achieve with exercise-based interventions and so the risk of bias has not been measured. This review adheres to the PRISMA guidelines and the PRISMA checklist is presented in the supplementary material.^76^

## Supplementary information

Supplementary tables and figures

## Data Availability

The authors declare that [the/all other] data supporting the findings of this study are available within the paper and the supporting references [and its supplementary information files].

## Supplementary information

**Supplementary Information** accompanies the paper on the *npj Microgravity* website (10.1038/s41526-019-0073-4).
