# Supplementary material for: A comparison of exercise interventions from bed rest studies for the prevention of musculoskeletal loss
Source: NPJ Microgravity. 2019 May 8;5:12. doi: 10.1038/s41526-019-0073-4 (PMC6506471; doi:10.1038/s41526-019-0073-4)
Supplement: Supplementary file 1 — Supplementary tables and figures [file 41526_2019_73_MOESM1_ESM.pdf]

Supplementary figure 1: Zero-gravity Locomotion Simulator. Photograph used with permission from Elsevier.[28]

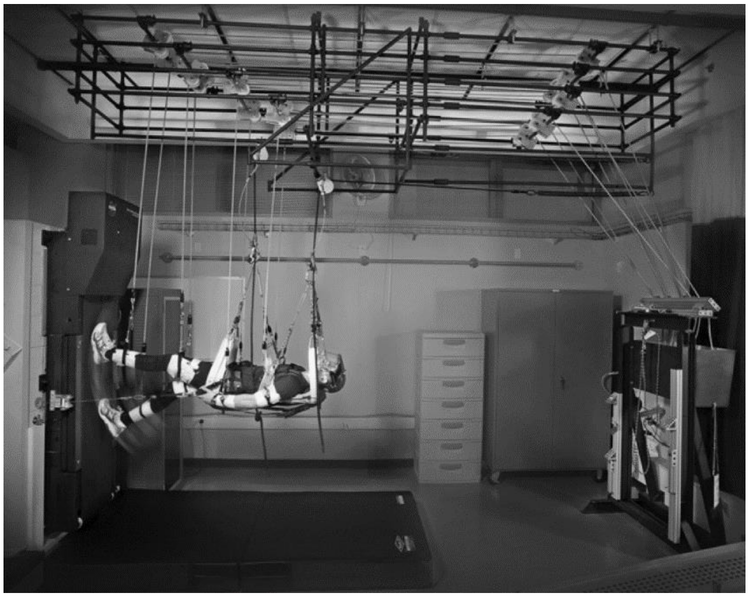

The zero-gravity locomotion simulator incorporates an off-loading suspension system to support a subject vertically while also integrating a treadmill machine.[28]

Supplementary figure 2: Resistive vibration exercise platform. Photograph used with permission from Elsevier.[29]

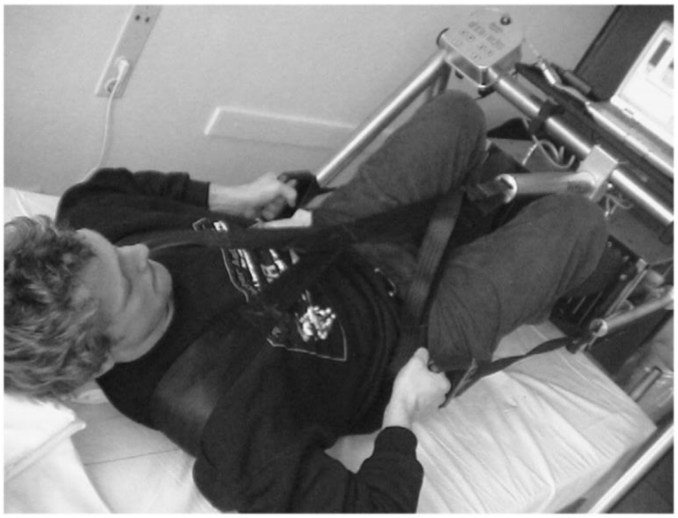

The subject is attached to the platform with belts and elastic springs to simulate microgravity. The eccentric rotation of masses provides the platform vibration.[29]

Supplementary figure 3: The principle of flywheel devices. Photograph used with permission from Elsevier.[78]

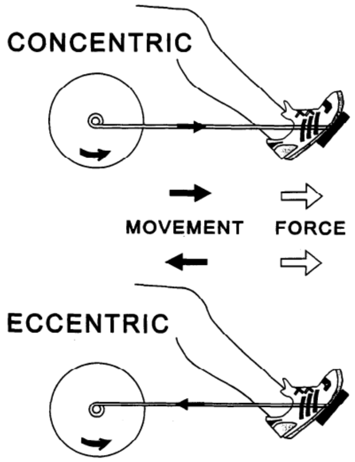

The force in the concentric pushing phase stores kinetic energy and eccentric phase slows the flywheel.

Supplementary figure 4: Lower body negative pressure (LBNP). Photograph used with permission from Elsevier.[71]

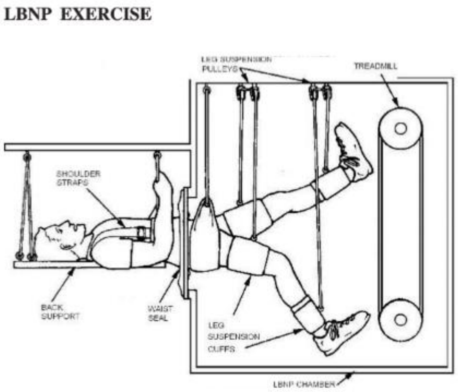

This figure illustrates LBNP treadmill exercise in a supine position with leg suspension and back support to simulate microgravity.

Supplementary table 1: MEDLINE search strategy

Database	Time Span	Search strategy	Results
**MEDLINE**	1946 to November 2018	1. Bone.mp or "Bone and Bones"/	865908
		2. Musculoskeletal.mp.	56676
		3. Osteoporosis/	41348
		4. 1 or 2 or 3	923817
		5. Bed rest.mp. or Bed Rest/	6870
		6. Head-down tilt.mp. or Head-Down Tilt/	2203
		7. 5 or 6	8419
		8. 4 and 7	897
		9. Limit 8 to English language	754
		10. Limit 9 to randomized controlled trial	53

The MEDLINE search was performed independently by two individual authors (NK, RK). The final search presented here was performed on 04/11/2018.

Supplementary table 2: Risk of bias assessment table for included studies.

	Randomisation (Selection bias)	Consistency between groups (Performance bias)	Incomplete outcome data (Attrition bias)	Selective outcome reporting (Reporting bias)	Degree to which compliance to intervention assessed
**Cavanagh PR 2016**	+	+	+	?	+
**Rittweger J 2010**	+	+	?	?	+
**Zwart SR 2007**	+	+	?	-	?
**Rittweger J 2005**	+	+	?	-	+
**Smith SM 2008**	+	+	+	-	?
**Shackelton 2004**	-	+	+	?	?
**Belavy 2011**	+	+	+	+	+
**Armbrecht 2011**	+	+	+	-	?

Risk of bias assessment table for included studies. Green represents low risk, red is high risk and orange is unclear risk of bias.
